# Sensor Technologies for Measuring Tongue Biomechanics Relevant to Swallowing: A Narrative Review

**DOI:** 10.3390/s26113453

**Published:** 2026-05-30

**Authors:** Cagla Kantarcigil, Loni Arrese, Sang Jun Kim, Isabella Gianakopoulos, Marina Bulazo, Min Ku Kim, Brittany N. Krekeler

**Affiliations:** 1 Department of Speech and Hearing Science, College of Arts and Sciences, The Ohio State University, 1070 Carmack Rd., Columbus, OH 43210, USA; arrese.1@osu.edu (L.A.); gianakopoulos.11@buckeyemail.osu.edu (I.G.); bulazo.2@buckeyemail.osu.edu (M.B.); 2 School of Mechanical Engineering, Hanyang University, 222 Wangsimni-ro, Seongdong-gu, Seoul 04763, Republic of Korea; tkdrabbit97@hanyang.ac.kr; 3 Department of Communication Sciences and Disorders, College of Allied Health Sciences, University of Cincinnati, 3225 Eden Avenue, ML 0528, Cincinnati, OH 45267, USA; krekelby@ucmail.uc.edu; 4 Departments of Otolaryngology and Neurology and Rehabilitation Medicine, College of Medicine, University of Cincinnati, 231 Albert Sabin Way, ML 0528, Cincinnati, OH 45267, USA

**Keywords:** dysphagia, tongue biomechanics, tongue pressure, intraoral sensors, swallowing assessment, dysphagia rehabilitation, biofeedback, sensor technologies, deglutition, pressure measurement

## Abstract

Tongue biomechanics are central to swallowing, yet commonly used clinical assessments provide limited insight into the forces and coordination underlying bolus propulsion. Sensor technologies have emerged to address this gap, but the literature remains fragmented across device classes, calibration approaches, and outcome definitions. This narrative review synthesizes sensor modalities used to characterize tongue biomechanics in dysphagia assessment and rehabilitation. A structured search of biomedical databases identified studies describing pneumatic, piezoelectric, strain gauge, capacitive, optical, and position-tracking systems. Across modalities, consistent physiological patterns are observed, including anterior-to-posterior pressure sequencing and task-dependent modulation with bolus properties. However, cross-study comparison is constrained by variability in sensor configuration, placement, and calibration, limiting the development of shared normative thresholds. To address this, we introduce a comparative maturity framework that situates modalities along a continuum from clinically established to proof-of-concept systems. Pneumatic and piezoelectric devices demonstrate the strongest evidence base and clinical integration, whereas capacitive and optical systems remain early-stage with minimal validation in patient populations. Position-tracking approaches provide complementary kinematic information but remain constrained by cost and ecological validity. Progress will require standardized calibration frameworks, harmonized protocols, and multimodal integration to support clinically interpretable metrics of tongue function.

## 1. Introduction

The tongue plays a central role in several vital physiological functions, including mastication, swallowing, and airway protection [1,2,3]. As a muscular hydrostat, it generates force and shape change through coordinated deformation of its intrinsic and extrinsic muscles, that are essential for bolus formation, manipulation, and anterior–posterior propulsion from the oral cavity into the pharynx [1,2,3]. During swallowing, the tongue also contributes to airway protection through its role in laryngeal vestibular closure [4,5]. These physiologic events require precise spatial and temporal activation of multiple muscle groups primarily innervated by the hypoglossal nerve, with integrated sensory feedback from trigeminal, glossopharyngeal, and vagal afferents, under the control of cortical, subcortical, and brainstem swallowing networks [1,2,3,6,7].

**Figure 1:**
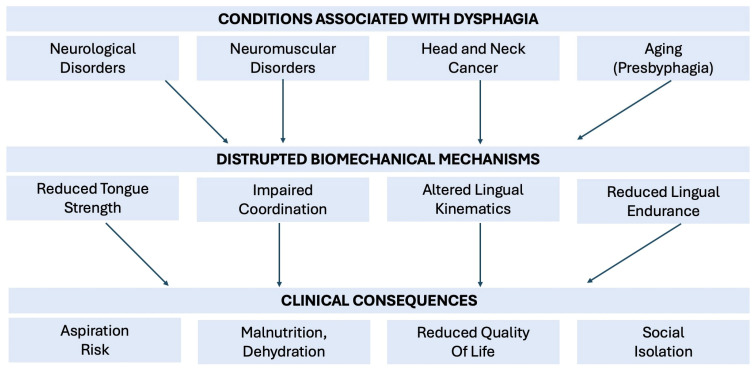
Dysphagia: Associated Conditions, Disrupted Mechanisms, and Clinical Consequences.

Disruptions to these neuromuscular and sensorimotor processes are a primary cause of swallowing disorders (dysphagia), which affect a substantial portion of the population and are particularly prevalent in individuals with stroke, Parkinson’s disease, amyotrophic lateral sclerosis, myasthenia gravis, head and neck cancer, and in aging populations (Figure 1) [8,9,10]. Across these conditions, pathology may arise from disruptions in central and peripheral neural control, muscle weakness or fatigue, and structural or sensory impairments affecting tongue function. These disease-specific mechanisms commonly manifest as reduced tongue pressure generation, delayed or discoordinated activation patterns, and altered lingual kinematics, leading to impaired bolus preparation and propulsion, increased oral residue, and reduced swallowing safety and efficiency. Dysphagia significantly impacts quality of life, leading to malnutrition, dehydration, and aspiration pneumonia, a potentially life-threatening complication [11,12,13]. Beyond the health-related consequences, dysphagia can cause social isolation due to difficulties with eating and participating in social activities that involve meals [14]. This can have a profound impact on an individual’s emotional well-being and overall quality of life.

Evaluation of dysphagia commonly involves both bedside assessments and imaging of swallowing structural movements, such as videofluoroscopic swallow studies (VFSS) or fiberoptic endoscopic evaluation of swallowing (FEES). While these methods provide dynamic visualization of bolus transit and airway safety, they do not directly quantify the underlying biomechanical forces driving swallowing, including tongue pressure generation, coordination, and endurance. Existing commercial sensor systems, such as the Iowa Oral Performance Instrument (IOPI^®^) and Tongueometer™, offer accessible means of quantifying tongue strength and endurance at the bedside. However, these devices rely on single-point pressure readings that do not reflect the spatial or temporal complexity of tongue movements during swallowing.

To address these limitations, a range of sensor technologies have emerged over the past several decades, including piezoelectric arrays, strain gauges, pneumatic bulbs, capacitive sensors, optical detectors, and kinematic tracking systems such as electromagnetic articulography. These platforms differ widely in their operating principles, anatomical coverage, sampling rates, and signal types. Some, such as pneumatic bulbs, have achieved clinical adoption [15,16], while others remain experimental. Although this diversity has advanced the characterization of tongue biomechanics, it has also contributed to a fragmented literature, with studies varying in sensor configurations, placement strategies, calibration procedures, outcome measures, and study populations. Much of this literature includes foundational studies in healthy adults as well as pilot studies and studies involving individuals with dysphagia, often with limited disease-specific characterization, which complicates cross-study comparison and diagnosis-specific interpretation. These challenges motivate the need for a synthesis of sensor modalities and their translational readiness.

This narrative review aims to describe tongue sensor technologies reported in the literature and summarize the operating principles, representative devices, and applications relevant to swallowing research and rehabilitation. The review considers technologies that capture complementary domains of tongue function, including strength generation, spatiotemporal coordination, and lingual kinematics, which have relevance across different etiologies. Rather than focusing on study-level outcomes, this review emphasizes how different sensor modalities capture distinct aspects of tongue biomechanics, their advantages and limitations, and their relative readiness for clinical translation. The goal is to clarify how different technologies characterize tongue function, identify methodological constraints, and highlight considerations for future development and clinical integration.

## 2. Scope of Review

This review adopts a narrative framework to synthesize and compare sensor modalities used to characterize tongue biomechanics relevant to swallowing. Literature was identified through structured searches of biomedical databases (PubMed, Scopus, Web of Science, CINAHL, EMBASE, and ProQuest Dissertations & Theses) conducted in 2024 and updated in February 2026, using keyword combinations related to tongue sensors, swallowing, pressure measurement, and biomechanics. Representative search terms and database search strategies are provided in Supplementary Material Table S1. Databases were searched from inception with no publication date restrictions. Reference lists of key articles were also screened to identify additional relevant studies.

This review does not aim to exhaustively catalog all published studies. Instead, it synthesizes representative examples across sensor modalities to compare underlying measurement principles and signal outputs, while identifying shared methodological challenges. Particular emphasis is placed on cross-cutting issues, including calibration, signal interpretation, and considerations related to clinical translation.

### 2.1. Eligibility Criteria

Eligible studies included peer-reviewed journal articles, dissertations, and conference abstracts involving human participants performing swallowing or related oral tasks (e.g., maximum tongue pressure). Both validation studies in healthy adults and clinical investigations involving individuals with dysphagia were included. No specific dysphagia etiologies were pre-specified for inclusion or exclusion, as the review was organized around sensor modalities rather than disease groups. Studies focused exclusively on speech-only applications, simulations, or animal models were excluded. Conference abstracts were considered when they described sensor platforms not otherwise available in full manuscript format and provided unique information relevant to modality characterization.

### 2.2. Study Selection

**Figure 2:**
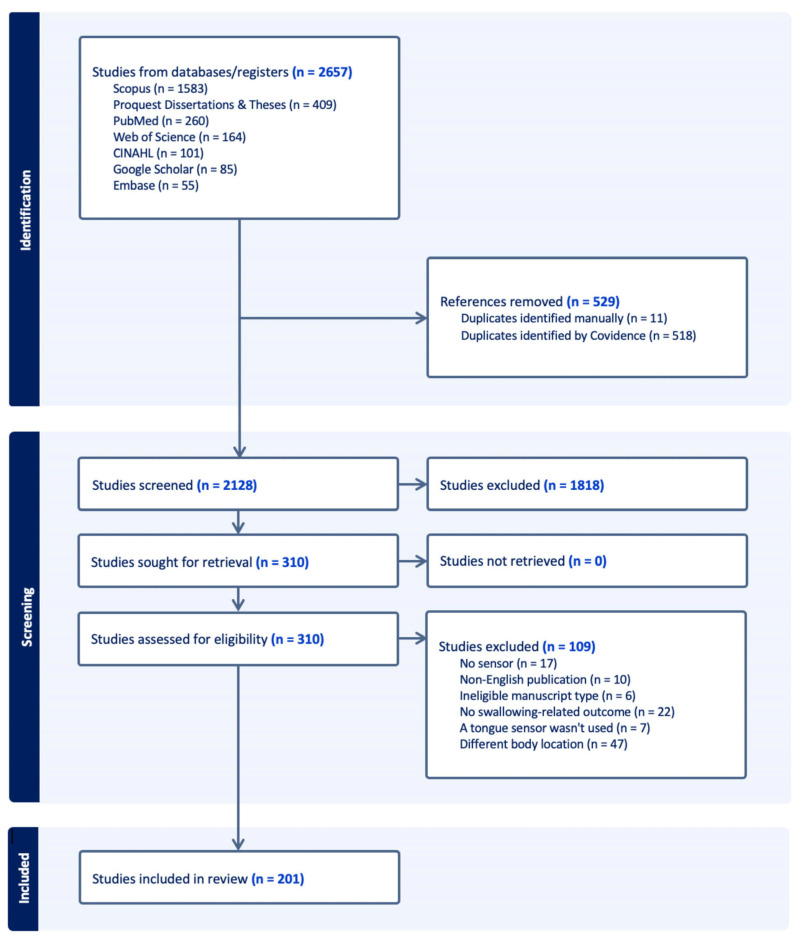
PRISMA Flow Diagram.

Search results were imported into Covidence for screening and duplicates were removed [17]. Titles and abstracts were screened against the predefined eligibility criteria, followed by full-text review of potentially eligible studies. Studies were excluded if they fell outside the scope of the review, including those not addressing tongue biomechanics relevant to swallowing or articles that did not provide sufficient information to characterize sensor modalities. A PRISMA-style flow diagram summarizes study identification, screening, eligibility assessment, and inclusion (Figure 2).

### 2.3. Data Organization and Synthesis

Included studies were examined to characterize sensor operating principles, representative device configurations, measurement outputs, methodological considerations, and reported applications. Information was organized using a structured extraction workflow to support comparison across sensor modalities.

For the purposes of this review, sensor technologies were classified according to their underlying measurement principles and signal outputs, rather than by device name or study context. Specifically, modalities were organized into six categories: (1) pneumatic systems, which measure tongue–palate pressure via deformation of air-filled bulbs; (2) piezoelectric systems, which convert mechanical deformation into electrical signals to enable high-resolution pressure mapping; (3) strain gauge systems, which estimate force through deformation of resistive elements; (4) capacitive systems, which detect tongue contact or proximity through changes in capacitance; (5) optical sensors, which use reflected light signals to detect tongue-palate interaction without direct force measurement; and (6) position tracking approaches, such as electromagnetic articulography, which characterize tongue motion and displacement.

## 3. Overview of Sensor Modalities

**Figure 3:**
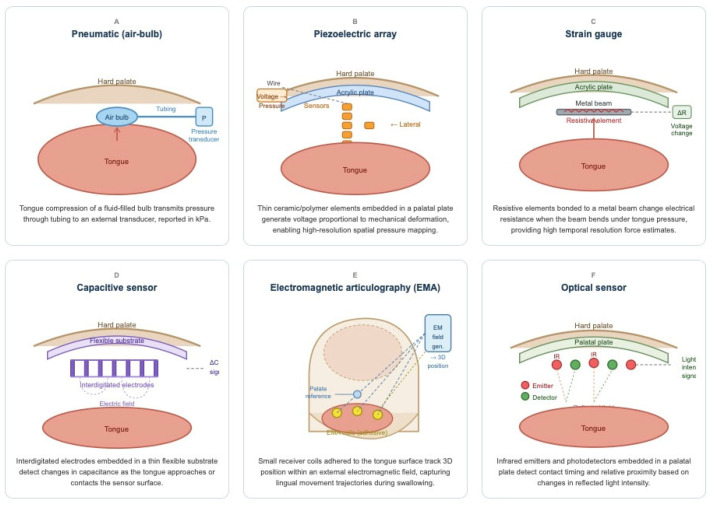
Schematic illustrations of tongue sensor modalities used to measure swallowing biomechanics.

This review identified a diverse set of sensor technologies that characterize tongue biomechanics. Devices range from well-established intraoral tools such as pneumatic air-bulb systems and piezoelectric palatal arrays that directly capture tongue–palate pressures, to extraoral motion-tracking techniques such as electromagnetic articulography (EMA) that quantify lingual movement patterns. In addition, exploratory modalities such as capacitive and optical sensors represent early-stage efforts to create thinner, more flexible arrays (Figure 3).

The evidence base was uneven across modalities, with the largest body of studies identified for pneumatic and piezoelectric systems, fewer studies for strain gauge and EMA approaches, and relatively limited reports for capacitive and optical systems (Table 1).

Across modalities, the central objectives have been to quantify tongue pressure generation, spatiotemporal sequencing, and lingual kinematics during swallowing, and to explore how these parameters change with aging, disease, and therapeutic intervention. Results are organized by sensor type, highlighting the underlying measurement principle, representative devices, applications, limitations, and future potential. Table 2 summarizes key differences across sensor modalities, including signal type, their primary measurement outputs, and the distinct biomechanical domains they capture.

## 4. Sensor Characteristics, Applications, and Limitations

### 4.1. Pneumatic (Air-Bulb) Sensors

Pneumatic (e.g., air-bulb) sensors are the most widely adopted tools for quantifying tongue–palate pressure in both experimental and clinical contexts. These systems convert compression of a compliant, fluid-filled bulb into changes in intra-bulb pressure, which are transmitted through narrow tubing to an external transducer and reported in kilopascals (kPa). Devices such as the Iowa Oral Performance Instrument (IOPI^®^), Tongueometer^TM^, and related systems are minimally invasive, relatively easy to implement, and have been broadly integrated into clinical assessment and rehabilitation protocols [18,19,20,21,22,23,24,25,26,27,28,29,30].

Pneumatic sensors have been evaluated across a wide range of populations, including healthy adults and diverse clinical groups such as stroke, neurodegenerative disease, and head and neck cancer, reflecting their relatively extensive use in both research and clinical contexts. Early implementations relied on single-bulb probes positioned against the hard-palate, most commonly just posterior to the incisive papilla, to quantify maximum isometric pressure (MIP) and swallowing-related pressures across a range of populations [31,32,33,34,35,36,37]. These studies established foundational relationships between tongue strength and swallowing function, including the concept of functional reserve, defined as the difference between MIP and pressures generated during swallowing [31,33,35,37,38,39,40]. Across studies, swallowing pressures were consistently submaximal relative to MIP, indicating that healthy swallowing operates within a reserve capacity rather than at maximal strength [33,35]. Furthermore, these early studies demonstrated that task-related factors, including bolus volume, viscosity, and swallowing maneuvers, systematically influence pressure generation, with effortful swallows producing higher pressures than typical swallows [35,41,42,43].

To improve spatial resolution, multi-bulb palatal arrays were developed, most notably three-bulb configurations aligned along the midline [44,45,46]. These systems enabled characterization of regional pressure profiles and revealed consistent anterior-to-posterior gradients in both timing and magnitude of tongue–palate pressure during swallowing [47,48,49]. Anterior sensors typically register earlier and higher peak pressures, whereas posterior sensors capture later, lower magnitude activity [48,50,51,52]. Studies using these arrays further demonstrated age- and disease-related differences, including reduced peak pressures and prolonged pressure rise time in older adults, consistent with diminished functional reserve with aging [23,51,53,54,55,56,57].

The Madison Oral Strengthening Therapeutic (MOST) (aka SwallowSTRONG) system further advanced pneumatic sensing by embedding five bulbs into custom-molded palatal plates [58,59,60,61,62]. This design moved beyond midline-only arrays by capturing multidirectional pressure distributions across anterior, posterior, and lateral contact sites. Data from these systems demonstrated consistent regional differences in pressure magnitude and temporal features, including anterior-dominant pressure generation and variations in time-to-peak across sensor locations [58,61]. Age-related effects were also evident, with reductions in MIPs and shallower pressure gradients observed in older adults. In addition, MOST-based analyses showed that tongue pressure generation varies across task conditions, with distinct profiles for isometric presses versus functional swallowing and modulation by bolus characteristics, supporting the view that lingual pressure is dynamically adapted to task demands rather than reflecting a fixed strength capacity [58].

Commercial devices such as the IOPI, Tongueometer, and JMS Digital Tongue Pressure Manometer (JMS) and IOPI illustrate both the clinical utility and measurement variability of pneumatic systems [25,27,32]. Although their outputs are strongly correlated, the Tongueometer often reports lower pressures than IOPI [25]. These device-specific offsets require separate normative datasets and complicate comparisons across laboratories.

Taken together, pneumatic sensors provide strong evidence that tongue pressure declines with age, correlates with dysphagia severity, and responds to therapeutic intervention [18,19,59,60,63]. Key limitations include variability linked to bulb size, tubing compliance, placement landmarks, and lip seal. Multi-bulb systems improve spatial coverage but may reduce comfort and ecological validity due to their bulky nature. Additionally, some configurations (e.g., the three-bulb midline array) are no longer commercially available, which limits reproducibility.

### 4.2. Piezoelectric Sensors

Piezoelectric sensors are among the most technically refined methods for recording tongue–palate contact pressures [64,65,66,67]. These devices rely on thin ceramic or polymer elements that generate voltage in response to mechanical deformation [66,67]. When embedded in palatal plates, they facilitate high-resolution mapping of pressure distributions across multiple intraoral sites [64,65,66,67]. Compared with pneumatic bulbs, piezoelectric systems offer faster response times and reduced compliance artifacts, facilitating more precise characterization of rapid pressure changes, though fabrication is more complex.

Evidence for piezoelectric arrays has been derived primarily from studies in healthy participants, with additional investigations in clinical populations, including neurodegenerative conditions such as ALS and Parkinson’s disease, although clinical representation remains more limited relative to healthy cohorts. The most widely used configuration has been a T-shaped palatal array with five channels arranged along the anterior–posterior midline and one lateral extension [41,68,69,70,71,72,73,74,75,76,77,78,79,80,81,82,83,84,85,86,87,88,89,90]. Sensors are typically a few millimeters in diameter and bonded to an acrylic palatal plate. Calibration is performed with known mechanical loads prior to intraoral use, but intraoral temperature and saliva can alter sensitivity and require periodic checks.

Studies with these arrays consistently show the anterior-to-posterior sequencing of tongue pressure [67,75,85,89,91]. The lateral channels typically record lower magnitudes and has been used to characterize regional differences in tongue-palate contact, including side-specific reductions in clinical populations [92]. Peak pressures scale with bolus viscosity and volume, while temporal measures such as duration and rise time adapt to task demands [79,81,90,93,94]. These systems have been particularly valuable for measuring spatiotemporal pressure patterns that are not captured by single-site measurements. Clinical work reports reduced magnitudes in older adults and in individuals with dysphagia due to stroke, Parkinson’s disease, or Muscular Dystrophy [80,82,83,87,92,95].

Limitations include the need for custom intraoral fabrication, burden associated with wiring, and challenges in sterilization for repeated clinical use. Although signal stability is generally high, sensitivity may be affected by intraoral conditions over time [64,65,66,67]. These practical constraints have limited widespread clinical adoption beyond research laboratories despite strong experimental validation.

Future directions include thinner, flexible piezoelectric films that can be laminated onto oral appliances or integrated into removable dental prostheses. Advances in wireless data transmission and biocompatible encapsulation may further support translation beyond laboratory settings.

### 4.3. Strain Gauge Sensors

Strain gauge systems are among the earliest methods for quantifying tongue pressure [96]. They use small resistive elements that change voltage when bent or compressed [96]. Gauges are typically bonded to thin metal beams embedded in acrylic palatal plates; when the tongue presses against the plate, the beam bends and the resulting strain is recorded electrically [96,97,98,99]. Sampling rates of 200–500 Hz provide excellent temporal resolution [96,97,98,99].

Unlike pneumatic systems, strain gauge recordings primarily reflect pressure at or after tongue–palate contact, providing limited insight into early bolus propulsion dynamics. Strain gauges were foundational in establishing tongue pressure patterns and sequencing during swallowing, including the anterior-to-posterior sequence and task-dependent modulation with viscosity [96,98,99]. Evidence for strain gauge–based systems has been derived predominantly from healthy cohorts, with limited representation of clinical populations, including a study that reported reduced tongue pressures in patients with oral cancers [97].

In general, strain gauge sensors provide high sensitivity, but the systems are fragile and subject to drift. Calibration requires repeated loading cycles with known weights, and readings can shift with intraoral temperature or adhesive degradation [96].

The main limitations are comfort and fabrication complexity. Plates are relatively bulky, wiring exits through the oral commissure, and sterilization is difficult. These factors have restricted strain gauge systems to laboratory settings [96,97,98,99]. Despite their decline in routine use, they remain an important benchmark for validating newer devices.

### 4.4. Capacitive Sensors

Capacitive sensors measure changes in capacitance, defined as the ability of a system to store electrical charge between two conductive surfaces. Capacitance varies as a function of both the separation between conductors and the dielectric properties of the intervening medium.

In these systems, arrays are formed by embedding interdigitated electrodes within an acrylic substrate, generating a localized electric field at the sensor surface [100]. As the tongue approaches or contacts the electrode, it alters both the effective distance to the conductive surface and the local dielectric constant (e.g., replacing air or saliva with soft tissue), producing a measurable change in capacitance [100]. These signals are then mapped to indices of contact or position rather than direct force measurements [101]. Calibration is typically performed under controlled conditions, although drift is common due to temperature shifts and saliva conductivity [102]. Arrays can be fabricated as thin and flexible structures, which improves intraoral comfort compared with bulkier systems [100,101].

Prototype arrays with multiple sensing channels provide localized information on contact timing and distribution [100,102]. Experimental studies demonstrate consistent special patterns of tongue-palate contact and task-depended modulation during oral tasks [102]. Evidence for capacitive sensor-based systems remains limited, with work largely confined to prototype evaluations controlled laboratory settings with healthy participants [100,101,102]. Translation to clinical dysphagia populations has not yet been studied systematically, and validation in patient cohorts remains minimal. This gap reflects both the novelty of the approach and the need for robust validation.

Advantages include comfort and the potential for high-density spatial sensing of tongue contact. Limitations include moisture sensitivity, signal noise, and fabrication complexity associated with intraoral deployment [102]. Advances in biocompatible materials, encapsulation strategies, and signal processing approaches may support broader application in clinical and ambulatory settings [101].

### 4.5. Position Tracking (EMA) Sensors

Position-tracking methods, such as electromagnetic articulography (EMA), quantify tongue kinematics rather than pressure, providing insight into spatial and temporal dynamics of lingual movement during swallowing and speech.

EMA systems use an external electromagnetic field generator placed near the head and small receiver coils attached to the tongue surface with dental adhesive. Coils (typically 2–3 mm in diameter) are secured with dental adhesive and connected to an external recording system, facilitating high-resolution tracking of three-dimensional tongue position and movement trajectories [103,104,105]. Calibration employs reference coils on the teeth or hard palate to correct for head movement [104].

Evidence for EMA systems has been derived primarily from studies in healthy participants, with only a limited number of investigations extending to clinical populations such as ALS. Accordingly, these approaches remain largely confined to laboratory-based research settings. In healthy adults, EMA has been used to characterize patterns of tongue movement, including anterior-to-posterior displacement and task-dependent timing during speech and swallowing [103,104,105]. In addition, emerging work using EMA demonstrates altered lingual kinematics in specific populations, including reduced range of motion, slower movement speeds, and changes in coordination during swallowing in individuals with amyotrophic lateral sclerosis [106].

EMA faces constraints on ecological validity as it requires adhesive coils and wiring that may alter natural swallowing, and the equipment is costly. However, this method can be valuable in mechanistic research and best interpreted alongside pressure-based measures.

### 4.6. Optical Sensors

Optical systems detect tongue–palate contact using light rather than mechanical coupling. Most prototypes embed infrared emitters and detectors in a palatal plate; contact or proximity alters reflected light, which is converted into timing and relative magnitude signals [107]. These systems detect relative changes in tongue proximity rather than calibrated force or pressure. Calibration can be performed under controlled conditions using known distances or reference positions, but signal quality may be influenced by saliva, mucosal reflectivity, and sensor alignment [107].

Evidence for optical sensor–based systems remains limited and has been derived primarily from feasibility and prototype studies in healthy participants. These studies map contact patterns during swallowing and speech and reproduce the anterior-to-posterior sequence seen with pressure-based devices [107]. However, signal variability is greater than in pressure-based systems, and outputs often reflect relative or binary contact states (contact/no contact) rather than continuous calibrated measurements. Published work has been limited to small feasibility samples in healthy adults [107], with little evidence from clinical populations. As a result, optical sensing remains at a proof-of-concept stage.

Key limitations include degradation with saliva accumulation, the need for precise emitter–detector alignment, and fabrication complexity associated with intraoral deployment. No commercially available platform currently exists. Future work may leverage miniaturized photodiodes and surface coatings to reduce noise and support clinical trials.

## 5. Comparative Maturity of Modalities

Comparative maturity was conceptualized in relation to four dimensions: (1) extent of clinical adoption, including degree of commercial availability or deployment; (2) strength and breadth of the validation evidence base; (3) evidence in patient populations; and (4) translational readiness, including usability and implementation constraints.

When compared across modalities, clear differences emerge in the extent to which each has been validated and translated toward clinical application (Table 3). Pneumatic and piezoelectric systems have the strongest evidence base, with validation across larger samples, established normative datasets across age groups, inclusion of clinical populations, and demonstrated utility in treatment studies [16,59,60,61,80,95]. They have demonstrated responsiveness to task manipulations and therapeutic interventions [18,19,20,23,59,60,79,98], and in the case of devices such as the IOPI, have entered routine clinical use.

Strain gauge systems, although critical in establishing foundational knowledge of tongue pressure dynamics, are largely restricted to laboratory use and serve primarily as reference tools against which newer systems are benchmarked [96,97,98,99]. Position-tracking techniques, such as EMA, provide valuable insights into kinematic mechanisms of swallowing but remain confined to specialized research environments due to cost, technical complexity, and ecological constraints [88,103,104,105,106,108].

By contrast, capacitive and optical sensors are at a proof-of-concept stage [100,101,102,107]. These systems offer the promise of thinner, more flexible arrays that minimize interference with natural swallowing; however, current evidence is largely limited to feasibility studies in healthy adults, and performance in clinical populations remains insufficiently characterized.

This gradient of maturity highlights central translational gaps. Established systems provide clinically accessible and reliable measures but offer limited insight into the spatial and temporal complexity of tongue biomechanics. In contrast, the experimental technologies capture richer biomechanical information but lack sufficient validation and clinical integration. Bridging this gap will require coordinated efforts to standardize measurement approaches, expand validation in patient populations, and integrate complementary sensing modalities.

## 6. Discussion

This review synthesized evidence across multiple classes of tongue sensor technologies, ranging from clinically established pneumatic devices to proof-of-concept capacitive and optical platforms. Across modalities, a central observation is that different technologies measure distinct dimensions of tongue function, including force, contact, and movement. These domains are complementary but not interchangeable, and each provides only a partial representation of swallowing biomechanics. While each modality has contributed distinct insights into tongue biomechanics and swallowing physiology, their collective evidence highlights both substantial progress and persistent challenges in achieving clinical translation. In this section, we interpret the findings across modalities, consider their implications for clinical assessment and therapy, evaluate methodological and engineering constraints, and outline priorities for future development.

### 6.1. Cross-Cutting Themes

Sensor modalities differ in design, application, and the dimension of tongue function that they measure, including force, contact, and movement. Despite these differences, several recurring challenges shape the overall field and determine the strength of the evidence base. These cross-cutting issues influence comparability across studies, reproducibility of findings, and ultimately the translational potential of tongue-sensor technologies.

Calibration and comparability remain persistent obstacles. Pneumatic bulbs vary according to bulb size, placement, and compliance; piezoelectric systems require voltage-to-pressure conversion using jigs or assumed contact areas; EMA demands careful field mapping and correction for head movement; and strain gauges are sensitive to the thickness and stiffness of their supporting plates. These design-specific factors create systematic differences that preclude pooled normative thresholds or simple cross-platform comparisons. The absence of shared calibration protocols means that results are often internally valid within a study but difficult to compare across devices or laboratories. Standardization of calibration methods, anatomical landmarks for placement, and transparent reporting of sensor characteristics would substantially improve comparability.

Measurement properties are also inconsistently reported. For pressure arrays, temporal measures such as rise time or duration typically demonstrate stronger reliability than peak magnitudes, which are more vulnerable to placement variability and device compliance. Evidence for convergent validity is strongest where posterior tongue pressures align with bolus clearance on imaging or where EMA trajectories replicate known kinematic patterns. Responsiveness (i.e., sensitivity to task manipulations or therapeutic change) is well established for pneumatic MIP/MSP and for piezoelectric measures under viscosity or effort manipulations. In contrast, newer modalities such as capacitive and optical systems lack longitudinal validation and have yet to establish whether they can detect clinically meaningful change. Importantly, these measurement properties are modality-dependent, reflecting differences in what each system is designed to capture.

Methodological constraints further limit synthesis. Most studies rely on small convenience samples, often of healthy young adults, which reduces generalizability. Bolus protocols vary widely in volume and viscosity, complicating comparisons across laboratories. Between-day reliability is rarely reported. Fixed palatal arrays pose additional risks of site mis-registration across different oral geometries, and many platforms remain confined to laboratory settings where ecological validity is limited. Collectively, these issues highlight the need for harmonized protocols and multicenter efforts to establish robust normative datasets.

Together, these cross-cutting themes emphasize that technical innovation alone is insufficient. Technologies that provide high spatial or temporal resolution often remain confined to laboratory settings, whereas clinically accessible systems prioritize simplicity and reproducibility at the expense of biomechanical detail. Progress will depend equally on methodological rigor, calibration standards, and collaborative approaches that allow meaningful aggregation of data across devices and laboratories.

### 6.2. Clinical Translation and Applications

The body of research reviewed here demonstrates that tongue sensor technologies have the potential to enhance how clinicians evaluate, monitor, and treat swallowing disorders. From a clinical perspective, sensor modalities can be differentiated by the specific biomechanical domains they quantify. Pneumatic systems are most directly suited to measuring tongue strength and endurance, making them clinically relevant for identifying reduced pressure generation, providing biofeedback during therapy, and monitoring response to tongue exercises. Piezoelectric arrays and multi-site pressure systems provide richer spatiotemporal information on tongue-palate contact and may be better suited for characterizing tongue–palate pressure sequencing, regional asymmetry, and task-dependent pressure modulation during swallowing. Strain gauge systems provide sensitive force measurements that have contributed to the mechanistic understanding of tongue biomechanics. Position-tracking approaches such as EMA capture lingual displacement, timing, and movement trajectories that may not be reflected in pressure measures alone. Emerging capacitive and optical systems are designed to capture tongue-palate contact patterns and proximity, with potential future relevance for home-based monitoring. Thus, these modalities capture complementary dimensions of tongue function and their clinical utility is best considered in relation to the specific physiological question being addressed.

Pneumatic air-bulb systems, including IOPI and Tongueometer, have already entered clinical practice because they are portable, inexpensive, and intuitive. They provide a direct, quantitative measure of tongue strength and endurance that clinicians can easily incorporate into diagnostic and therapeutic protocols. Importantly, these measures have been linked to dysphagia severity, aspiration risk, and rehabilitation outcomes, supporting their role as clinically meaningful indicators of swallowing function [18,19,59,60]. However, these tools capture only peak pressure values in isolated locations and thus oversimplify tongue biomechanics. Multi-site pneumatic platforms provide richer spatial resolution by capturing pressure patterns across anterior, mid, posterior and lateral palate regions [32,48,49,51,58,61]. However, their use requires custom molding, which limits practicality outside controlled research environments.

Piezoelectric arrays and strain-gauge devices, though mostly confined to research laboratories, offer finer spatiotemporal detail and a clearer picture of tongue–palate contact during swallowing. These systems have been particularly valuable for identifying patterns of compensation and altered timing in aging and disease [75,80,82,83,91,92,95,109,110]. Yet these systems are not widely accessible outside research settings, limiting their impact on day-to-day clinical care. A central challenge for clinical translation is not measurement capability, but whether these systems can move beyond research settings and be used by clinicians and patients in routine care.

Experimental capacitive and optical systems introduce thinner, more flexible sensing architectures that reduce interference with natural swallowing [100,101,102,107]. However, these platforms remain in proof-of-concept stage and are not yet sufficient as standalone tools for clinical assessment. Capacitive sensors can be integrated into flexible palatal plates, while optical sensors can detect contact without direct mechanical pressure. If validated in patients, these could offer comfortable long-term monitoring and assessment in more naturalistic settings, including outside the clinic. At present, however, evidence remains limited to small feasibility studies, predominantly in healthy participants, which constrains immediate clinical application [100,101,102,107]. Moving toward clinical adoption will require large-scale normative datasets, stratified by age, sex, and clinical population, to establish meaningful reference values.

### 6.3. Research Implications

From a research perspective, tongue sensors have been commonly used to study the biomechanics of normal and disordered swallowing. Pneumatic sensors confirmed the concept of “functional reserve,” showing that maximum swallowing pressures are consistently below maximum isometric pressures [44,58,111]. Piezoelectric arrays revealed anterior-to-posterior pressure sequencing, bolus-dependent modulation, and task-related adaptations in timing and magnitude [75,80,82,83,91,92,95,109,110]. Strain gauges, though less common today, established many foundational observations about pressure magnitudes and durations [96,98].

Yet methodological inconsistencies remain a major barrier to synthesis across studies. Studies vary widely in terms of bolus size, consistency, posture, and number of trials, making direct comparisons difficult. Placement landmarks are not standardized: anterior bulbs may be positioned just posterior to incisors in some studies but further back in others, resulting in systematic differences in recorded pressure values. Sampling frequencies also differ with pneumatic systems typically operating at lower sampling rates than piezoelectric arrays, leading to differences in temporal resolution. Reporting practices are equally variable, with some studies emphasizing peak pressures, others focusing on rise time or contact duration, and others reporting derived indices such as functional reserve or asymmetry ratios.

Future research should prioritize standardized experimental protocols, ideally through consensus guidelines similar to those developed for videofluoroscopic swallowing studies. Establishing common bolus stimuli, placement conventions, and reporting metrics would facilitate pooling of data across centers. This is particularly important for clinical populations where sample sizes are inherently small (e.g., ALS, head and neck cancer). In addition, greater emphasis on between-session reliability and cross-device comparability will be essential for building robust, generalizable datasets. A coordinated multicenter approach could accelerate the creation of large normative and patient databases, which in turn would enable predictive modeling of swallowing impairments and responses to therapy.

### 6.4. Engineering Considerations

From an engineering standpoint, every modality carries unique trade-offs between measurement fidelity, usability, and durability. Pneumatic devices are robust, inexpensive, and easy to calibrate, but their accuracy is limited by bulb geometry, wall compliance, and tubing length. Piezoelectric sensors provide high fidelity but require careful voltage-to-pressure calibration and stable contact between rigid plates and the tongue surface, which may alter natural kinematics. Strain gauges are sensitive but can be bulky, with plate stiffness that constrains physiological tongue movement. Capacitive systems are flexible and comfortable, but prone to drift and cross-talk. Optical sensors avoid mechanical loading entirely but are vulnerable to saliva, light occlusion, and intraoral humidity. These modality-specific constraints highlight that improvements in one domain (e.g., flexibility or sensitivity) often introduce new sources of error or instability.

Wireless data transmission remains a major unmet need. Nearly all systems rely on tethered cables leading outside the mouth, which interfere with natural chewing and swallowing. Advances in flexible printed electronics, Bluetooth, and biocompatible encapsulation could facilitate the development of fully intraoral, untethered devices. Battery life, miniaturization, and waterproofing are engineering challenges that must be solved in tandem. Equally important is device durability, as repeated cycles of compression and prolonged exposure to moisture can degrade many sensors over time. Incorporating novel materials such as PEDOT:PSS conductive polymers or graphene composites could improve resilience while preserving thin, flexible form factors.

Calibration remains a central engineering challenge across modalities. Piezoelectric devices need voltage-to-force conversion, pneumatic bulbs depend on stable reference pressures, and capacitive systems are susceptible to temporal drift. Without consistent calibration methods, sensor outputs may vary across sessions, devices, and laboratories, limiting reproducibility and comparability. Engineering solutions such as built-in reference channels, self-calibrating algorithms, or standardized in-mouth fixtures could significantly improve measurement reliability and facilitate cross-platform comparison.

### 6.5. Commercialization Pathways

The translational trajectories of tongue-sensor devices illustrate why some technologies progress toward clinical adoption while others remain confined to research settings. The Iowa Oral Performance Instrument (IOPI^®^), for example, achieved widespread clinical use because it was simple, portable, and required minimal training. Its outputs were reported in familiar units (kPa), and normative values were quickly established, facilitating integration into both clinical practice and research. By contrast, more complex multi-site platforms generated rich data but proved costly, cumbersome, and less compatible with routine workflows, which limited their uptake despite their technical sophistication. These contrasting trajectories demonstrate that clinical adoption depends not only on technical capability, but also on usability, interpretability, and integration into existing care pathways. Key translational barriers across tongue sensor modalities are summarized in Table 4, organized by engineering, clinical, and validation domains.

Future commercialization will depend on identifying applications where sensor-based metrics provide actionable clinical value. Devices that can be deployed easily in clinical and home settings, provide interpretable outputs for clinicians and patients, and demonstrate responsiveness to therapeutic interventions are most likely to achieve widespread adoption. In particular, systems that deliver real-time feedback, support remote monitoring, and align with telehealth models may offer the greatest near-term impact. Real-time biofeedback, compatibility with telehealth delivery, and seamless integration with existing diagnostic workflows represent particularly promising avenues.

Regulatory approval processes represent an essential step toward translation. Devices intended for diagnostic or therapeutic decision-making will require FDA clearance (or equivalent), which necessitates rigorous validation in patient populations. Academic prototypes often fail to progress because they are tested only in small samples of healthy adults and never undergo large-scale clinical trials. Early consideration of regulatory pathways, including study design, endpoints, and usability requirements, will be critical for successful translation. Building pathways to commercialization will require industry partnerships, multicenter collaborations, and early attention to usability and manufacturability alongside scientific validation.

### 6.6. Future Directions

Looking ahead, several converging trends suggest where the field is headed. First, multimodal sensing is likely to dominate. A single modality rarely captures the full complexity of swallowing; pressure alone cannot reveal tongue kinematics, and motion tracking alone cannot indicate bolus propulsion forces. Future systems will likely integrate complementary sensing modalities to capture force, contact, and movement within a unified framework. Combining pressure arrays with accelerometers, acoustic sensors, or wearable neck patches could yield a more comprehensive and physiologically meaningful representation of swallowing.

Recent advances in wearable and flexible sensor technologies for swallowing assessment further illustrate this broader trend toward continuous, less obtrusive monitoring beyond the clinical environment. Epidermal and skin-interfacing devices have been developed to capture swallowing-related physiological signals, including muscle activity, laryngeal motion, and acoustic or accelerometric signatures, using compliant materials and stretchable electronic architectures [112,113,114]. Flexible and self-powered sensing platforms, including triboelectric nanogenerator-based systems, also demonstrate the potential of soft, conformal materials and integrated signal processing for physiological monitoring [115]. However, most of these approaches have been applied to external body sites and capture indirect correlates of swallowing rather than intraoral tongue biomechanics [116]. Extending these design principles to tongue-sensor technologies will require adaptation to the mechanical, spatial, and moisture-related constraints of the oral cavity, while preserving the ability to quantify force generation, spatiotemporal coordination, and lingual kinematics.

Second, machine learning and signal analytics hold promise for extracting clinically meaningful indices from high-dimensional datasets. Piezoelectric arrays, for example, generate rich spatiotemporal maps that are difficult to interpret by visual inspection alone. Machine learning models trained on large datasets could identify subtle biomarkers predictive of aspiration risk or therapeutic response. However, these approaches will depend on the availability of standardized, high-quality datasets to ensure generalizability and avoid overfitting.

Third, there is growing interest in home-based monitoring. Dysphagia is a chronic condition that evolves over time, but current assessments are episodic, conducted only in clinics. Miniaturized, wireless, and comfortable devices would enable continuous monitoring of swallowing in natural environments, providing richer data for both research and patient care. This shift toward longitudinal monitoring may be particularly valuable for tracking disease progression and treatment response.

Finally, patient-centered design must be prioritized. Many prototypes fail because they are uncomfortable, intrusive, or incompatible with dentures and palatal prostheses. Future devices must balance measurement fidelity with wearability and usability to achieve sustained adoption. Co-design with patients and clinicians emphasizing usability, hygiene, and comfort will be essential for successful translation.

## 7. Conclusions

In sum, tongue sensor technologies span a spectrum from mature, widely adopted pneumatic bulbs to experimental capacitive and optical platforms. Each modality offers unique insights into swallowing biomechanics but also carries distinct limitations, reflecting the different dimensions of tongue function they are designed to measure. For clinical adoption, the central challenges remain calibration, standardization, and usability. For research, established protocols and multicenter collaboration are needed to build the datasets that will unlock predictive modeling. For engineering, advances in wireless transmission, flexible materials, and multimodal integration are the next frontier. Future progress will depend on integrating complementary sensing approaches to capture force, contact, and movement in a coordinated manner. If these challenges can be met, tongue sensors have the potential to move beyond research laboratories and become integral to the assessment and rehabilitation of swallowing disorders.

## Figures and Tables

**Table 1:** Sensor Modality Distribution Across Included Studies.

Sensor Modality	Number of Studies
Pneumatic	114
Piezoelectric	74
Strain gauge	4
Electromagnetic articulography	5
Capacitive	3
Optical	1

**Table 2:** Overview of Tongue Sensor Technologies: Primary Measurement Principles and Biomechanical Domains.

Sensor Modality	Signal Type	Primary Measurement Output	Biomechanical Domain Captured
Pneumatic (Air-bulb) [18,19,20,21,22,23,24,25,26,27,28,29,30,31,32,33,34,35,36,37,38,39,40,41,42,43,44,45,46,47,48,49,50,51,52,53,54,55,56,57,58,59,60,61,62,63]	Intra-balloon pressure converted pressure (kPa)	Pressure (kPa) reflecting tongue-palate force at discrete locations	Force (pressure)
Piezoelectric [64,65,66,67,68,69,70,71,72,73,74,75,76,77,78,79,80,81,82,83,84,85,86,87,88,89,90,91,92,93,94,95]	Voltage generated by mechanical deformation of ceramic/polymer elements	High-resolution pressure mapping derived from voltage generated by mechanical deformation	Force (spatiotemporal pressure)
Strain Gauge [96,97,98,99]	Voltage changes from resistive elements bending or compressing	Pressure/force estimation based on resistive deformation of structural elements	Force
Capacitive [100,101,102]	Capacitance changes occurring when the tongue contacts or approaches electrodes	Contact/proximity detection based on changes in capacitance.	Contact/proximity
Position Tracking (Electromagnetic articulography) [103,104,105,106]	Kinematics (3D position tracking)	Three-dimensional tongue motion trajectories	Motion (kinematics)
Optical [107]	Reflected light changes indicating timing and relative magnitude of contact	Contact timing and relative magnitude based on reflected light changes	Contact

**Table 3:** Comparative Maturity Spectrum.

Maturity Level	Sensor Modalities	Status and Evidence Base
Stage 4: Most Clinically Established	Pneumatic (Air-bulb)	Most mature. Widely adopted in routine clinical use (e.g., IOPI^®^, Tongueometer™). Extensive normative datasets across age groups and proven responsiveness to therapy.
Stage 3: Advanced Research Use	Piezoelectric; Position Tracking (Electromagnetic Articulography)	Technically advanced. Provides high-resolution mapping (piezoelectric) or kinematic (EMA) data. Robust evidence in research settings, but limited clinical adoption due to cost, complexity, and the need for custom dental impressions.
Stage 2: Foundational/Legacy Systems	Strain Gauges	Legacy technology. Foundational technology that established early pressure patterns and normative data. Now largely restricted to laboratory use as a reference tool for benchmarking newer devices.
Stage 1: Proof-of-Concept	Capacitive and Optical Sensors	Experimental. Offers promise for thin, flexible, and comfortable arrays. Currently limited to feasibility studies in healthy adults; performance in clinical populations remains largely untested.

**Table 4:** Key translational barriers across tongue sensor modalities.

Sensor Modality	Technical and Engineering Constraints	Clinical and Practical Constraints	Evidence and Validation Gaps
**Pneumatic (Air-bulb) [18,19,20,21,22,23,24,25,26,27,28,29,30,31,32,33,34,35,36,37,38,39,40,41,42,43,44,45,46,47,48,49,50,51,52,53,54,55,56,57,58,59,60,61,62,63]**	Accuracy is influenced by bulb geometry, wall compliance, and tubing length.	Variability in anatomical placement and dependence on adequate lip seal.	Device-specific offsets (e.g., IOPI vs. JMS Digital Tongue Pressure Manometer) limit cross-platform comparability and the development of shared normative datasets.
**Piezoelectric [64,65,66,67,68,69,70,71,72,73,74,75,76,77,78,79,80,81,82,83,84,85,86,87,88,89,90,91,92,93,94,95]**	Variability in anatomical placement and dependence on adequate lip seal.	Requires custom dental impressions and acrylic plates; difficult to sterilize for repeated use.	High cost and complexity limit use to specialized research environments rather than daily care.
**Strain Gauge [96,97,98,99]**	Susceptible to signal drift and mechanical fragility; performance may degrade with repeated use.	Bulky form factor and external wiring that exits the mouth, interfering with natural swallow function.	Largely considered legacy technology; with limited ongoing commercial development or clinical integration.
**Capacitive [100,101,102]**	Highly sensitive to moisture and saliva conductivity; prone to signal noise and cross-talk.	Fabrication complexity and current lack of biocompatible coatings for long-term use.	Evidence largely limited to feasibility studies in healthy participants; minimal data in clinical dysphagia populations.
**Position Tracking (Electromagnetic Articulography) [103,104,105]**	Requires complex magnetic field calibration and correction for head movement.	Adhesive coils and tethered wiring may alter natural swallowing mechanics and reduce ecological validity.	High cost and technical complexity limit use to specialized research settings; limited clinical translation.
**Optical [107]**	Signal degradation due to saliva accumulation, light occlusion, and intraoral humidity.	No commercially available platform exists; requires precise emitter–detector alignment.	Proof-of-concept stage only; evidence is limited to very small feasibility samples of healthy adults.

## Data Availability

No new data were created or analyzed in this study. All data supporting the findings of this review are derived from previously published studies and are cited within the article.

## Supplementary Materials

The following supporting information can be downloaded at: https://www.mdpi.com/article/10.3390/s26113453/s1, Table S1: Representative search terms and database search strategies.

## Abbreviations

The following abbreviations are used in this manuscript:

EMA	Electromagnetic articulography
FEES	Fiberoptic endoscopic evaluation of swallowing
IOPI	Iowa Oral Performance Instrument
JMS	JMS Digital Tongue Pressure Manometer
MIP	Maximum isometric pressure
MOST	Madison Oral Strengthening Therapeutic
MSP	Maximum swallow pressure
VFSSs	Videofluoroscopic swallow studies
