# Supplementary material for: Sensor Technologies for Measuring Tongue Biomechanics Relevant to Swallowing: A Narrative Review
Source: Sensors (Basel). 2026 May 30;26(11):3453. doi: 10.3390/s26113453 (PMC13258970; doi:10.3390/s26113453)
Supplement: Supplementary file 1 [file sensors-26-03453-s001.zip › sensors-4265134-supplementary.pdf]

Table S1. Representative search terms and database search strategies

Database	Search Terms
**PubMed**	Wearable Electronic Devices"[Majr]) OR ("Manometry"[Mesh]) OR (textile electronics) OR (wearable sensors) OR (pressure sensor*) OR (sensing probes) OR (sensor sheet)) AND ((deglutition) OR (deglutition disorders) OR (dysphagia) OR (pharyngeal diseases) OR (pharyn*) OR (nasopharyn*) OR (oropharyn*) OR (esophageal sphincter) OR (velopharyn*) OR (tonsil*) OR (swallow*))) AND ((tongue))
**EMBASE**	(((wearable AND electronic AND devices:ab,ti OR manometry:ab,ti OR 'textile'/exp OR textile) AND electronics:ab,ti OR wearable) AND sensor:ab,ti OR 'pressure'/exp OR pressure) AND sensor*:ab,ti OR 'sensing'/exp OR sensing) AND probes:ab,ti OR 'sensor'/exp OR sensor) AND sheet:ab,ti AND ((((deglutition:ab,ti OR 'deglutition'/exp OR deglutition) AND disorders:ab,ti OR dysphagia:ab,ti OR pharyngeal) AND diseases:ab,ti OR pharynx:ab,ti OR nasopharyn*:ab,ti OR oropharyn*:ab,ti OR esophageal) AND sphincter:ab,ti OR velopharyn*:ab,ti OR swallow*:ab,ti OR tonsil*:ab,ti) AND 'tongue':ab,ti
**CINAHL PLUS with FULL TEXT**	(wearable electronic devices OR manometry OR textile electronics OR wearable sensors OR pressure sensor* OR sensing probes OR sensor sheet) AND (deglutition OR Deglutition Disorders OR dysphagia OR Pharyngeal Diseases OR Pharynx OR Nasopharyn* OR Oropharyn* OR esophageal sphincter OR velopharyn* OR swallow* OR tonsil*) AND tongue
**SCOPUS**	TITLE-ABS-KEY (wearable AND electronic AND devices) OR (manometry) OR (textile AND electronics) OR (wearable AND sensors) OR (pressure AND sensor*) OR (sensing AND probes) OR (sensor AND sheet) AND (deglutition) OR (deglutition AND disorders) OR (dysphagia) OR (pharyngeal AND diseases) OR (pharynx) OR (nasopharyn*) OR (oropharyn*) OR (esophageal AND sphincter) OR (velopharyn*) OR (swallow*) OR (tonsil*) AND (tongue) AND (EXCLUDE (SUBJAREA , "ECON") OR EXCLUDE (SUBJAREA , "ENER") OR EXCLUDE (SUBJAREA , "BUSI") OR EXCLUDE (SUBJAREA , "EART") OR EXCLUDE (SUBJAREA , "MATH") OR EXCLUDE (SUBJAREA , "VETE") OR EXCLUDE (SUBJAREA , "ENVI") OR EXCLUDE (SUBJAREA , "IMMU") OR EXCLUDE (SUBJAREA , "CENG") OR EXCLUDE (SUBJAREA , "MATE") OR EXCLUDE (SUBJAREA , "PHYS") OR EXCLUDE (SUBJAREA , "COMP") OR EXCLUDE (SUBJAREA , "ARTS") OR EXCLUDE (SUBJAREA , "PSYC") OR EXCLUDE (SUBJAREA , "NURS") OR EXCLUDE (SUBJAREA , "PHAR") OR EXCLUDE (SUBJAREA , "CHEM") OR EXCLUDE (SUBJAREA , "SOCI") OR EXCLUDE (SUBJAREA , "AGRI") OR EXCLUDE (SUBJAREA , "NEUR") OR EXCLUDE (SUBJAREA , "ENGI") OR EXCLUDE (SUBJAREA , "BIOC")) AND (EXCLUDE (EXACTKEYWORD , "Animal") OR EXCLUDE (EXACTKEYWORD , "Animal Experiment"))
**Web of Science (Core Collection)**	"wearable sensors" OR “pressure sensor*” OR “sensing probes” OR “sensor sheet” OR “textile electronics” OR “wearable electronics” OR “manometry” (Topic) AND (“deglutition" [MeSH Terms] "Deglutition Disorders"[MeSH Terms] OR “dysphagia” OR “Pharyngeal

	Diseases"[MeSH Terms] OR "Pharynx"[MeSH Terms] OR "Nasopharyn*"[MeSH Terms] OR "Oropharyn*"[MeSH Terms]) OR "(esophageal sphincter, upper"[MeSH Terms] OR "esophageal sphincter, lower"[MeSH Terms] OR, “velopharyn*” OR (swallow*) OR (tonsil*) (Topic) AND tongue (Topic)
**ProQuest Dissertations & Theses**	("wearable electronic devices" OR manometry OR "textile electronics" OR "wearable sensors" OR "pressure sensor*" OR "sensing probes" OR "sensor sheet") AND (deglutition OR "deglutition disorders" OR dysphagia OR "pharyngeal diseases" OR pharynx OR "nasopharyn*" OR oropharyn* OR "esophageal sphincter" OR velopharyn* OR swallow* OR tonsil*) AND tongue
